# An Internet of Things System for Underground Mine Air Quality Pollutant Prediction Based on Azure Machine Learning

**DOI:** 10.3390/s18040930

**Published:** 2018-03-21

**Authors:** ByungWan Jo, Rana Muhammad Asad Khan

**Affiliations:** Department of Civil and Environmental Engineering, Hanyang University, 222 Wangsimni-ro, Seongdong-gu, Seoul 04763, Korea; joycon@hanmail.net

**Keywords:** underground coal mines, internet-of-things, azure machine learning, artificial neural network, mine environment index

## Abstract

The implementation of wireless sensor networks (WSNs) for monitoring the complex, dynamic, and harsh environment of underground coal mines (UCMs) is sought around the world to enhance safety. However, previously developed smart systems are limited to monitoring or, in a few cases, can report events. Therefore, this study introduces a reliable, efficient, and cost-effective internet of things (IoT) system for air quality monitoring with newly added features of assessment and pollutant prediction. This system is comprised of sensor modules, communication protocols, and a base station, running Azure Machine Learning (AML) Studio over it. Arduino-based sensor modules with eight different parameters were installed at separate locations of an operational UCM. Based on the sensed data, the proposed system assesses mine air quality in terms of the mine environment index (MEI). Principal component analysis (PCA) identified CH_4_, CO, SO_2_, and H_2_S as the most influencing gases significantly affecting mine air quality. The results of PCA were fed into the ANN model in AML studio, which enabled the prediction of MEI. An optimum number of neurons were determined for both actual input and PCA-based input parameters. The results showed a better performance of the PCA-based ANN for MEI prediction, with *R*^2^ and RMSE values of 0.6654 and 0.2104, respectively. Therefore, the proposed Arduino and AML-based system enhances mine environmental safety by quickly assessing and predicting mine air quality.

## 1. Introduction

The harsh and confined working conditions in underground coal mines (UCMs), have led to the listing of the mining industry as the most dangerous profession [1]. In recent years, the adoption of sophisticated regulations has greatly reduced mine accidents; yet, hundreds of miners lose their lives every year. According to the Mine Safety and Health Administration (MSHA), faulty equipment, negligence of labor towards explosions, structure failure, and gas accumulation are the most common causes of underground mine accidents [2]. During the economic year of 2014, in the salt range coal mine in Punjab, Pakistan, more than 35% of accidents occurred due to the accumulation of toxic gases [3]. Therefore, for the safety of workers and the mine itself, it is extremely important to continuously and accurately monitor the mine environment.

In recent years, advancements in the fields of wireless sensor network (WSNs), radio frequency identification (RFID), and cloud computing have led the way toward the development of internet of things (IoT) in the areas of Smart Grids, e-health services, home automation, and environment monitoring. Similarly, with reference to UCMs, the introduction of WSNs and the concept of smart monitoring have enabled accurate, cost-effective, and reliable real-time environmental monitoring. These technologies not only allow monitoring of inaccessible places but also assist in automatic event detection, control, and remote information sharing [4]. For instance, an efficient warning system [5] based on WSN has been introduced for early fire detection in a bord-and-pillar coal mine with capabilities of determining fire direction. Zhang et al. [6] integrated WSN and a cable monitoring system for multi-parameter environmental monitoring at Shangwan Coal Mine, Erdos, China. This system has two operational modes—periodic inspection and interruption service—and its feasibility for real mining conditions has been proven. Similarly, Issac [7] integrated WSN and ambient intelligence to detect the presence of toxic gases in a mine environment and to respond accordingly. This system introduced mobile sensing for the safety of underground mines. Recently, Jo and Khan [8] introduced an open-source, cost-effective, Arduino-based IoT system for early-warning and event reporting in UCMs. Another comprehensive and energy efficient monitoring system [9] based on WSN for early-event detection has been proposed to enhance safety in the challenging environment of UCMs. In addition to all these, detailed literature, focusing on the implementation of WSNs for mine environment monitoring, can be found in [9].

Along with accurate and continuous monitoring, mine air quality assessment and prediction can play a vital role in enhancing mine safety. This would not only reduce miners’ contact with bad air but also would allow efficient control of mine ventilation. However, the complex and non-linear behaviors of air quality variables are beyond the capabilities of a simple mathematical prediction formula [10]. In this regard, various statistical tools, such as ARIMA, multiple linear regression (MLR), artificial neural network (ANN), fuzzy time series, and principal component analysis (PCA) have shown highly satisfactory results for accurately assessing air quality and forecasting pollutant concentrations [11,12,13,14]. Concentrations of toxic gases are the major controlling factors of air quality; therefore, determination of the type and number of pollutants present in a specific environment is of extreme importance. In this regard, the statistical tool PCA has shown a high capability for identifying influencing pollutants, and ANN has enabled more accurate predictions. Therefore, in the harsh environment of UCM, a hybrid approach of PCA and ANN may be able to efficiently identify pollutants, along with giving accurate predictions.

This study aims to develop a reliable, cost-effective, and efficient IoT system for air quality monitoring and assessment in underground mines with the additional features of pollutant identification and air quality forecasting, based on Azure machine learning (AML) cloud computing. This study uses economical clones of Arduino-based sensor modules to monitor eight different air quality parameters (temperature, humidity, CO_2_, CH_4_, CO, SO_2_, H_2_S, and NO_2_), applies PCA to identify the most influential variables, and uses ANN to define a prediction model for mine air quality. The key contributions of this study are as follows:

(1) Arduino-based sensor modules for environmental monitoring in UCM,
(2) Mine environment index (MEI),
(3) Use of AML platform for mine air quality prediction,
(4) Identification of the most influential pollutants present in the mine environment and modelling of mine air quality for an accurate prediction of MEI.

The remainder of this paper is structured as follows: Section 2 summarizes the literature review related to PCA and ANN. Section 3 describes the architecture of the proposed system, discusses the selection of sensors, and introduces the AML. Section 4 focuses on a hybrid approach that integrates PCA and ANN, and it defines the parameters for model building. Section 5 and Section 6 illustrate the sensors’ calibration and system installation. The results of the proposed system are discussed in Section 7. Finally, conclusions are drawn in the last section, followed by the limitations and future considerations.

## 2. Literature Review

In recent decades, various scientific studies [15,16] have used multivariate statistical approaches, such as cluster analysis (CA), PCA, factor analysis (FA), and discriminant analysis for solving environmental and air quality issues. However, based on the eigenvalues solution, PCA is the most prevailing and simplest technique. Specifically, in air quality problems, it has been used alone or in combination with other approaches. For instance, [17,18] used PCA and CA to figure out the seasonal variations and spatial distribution of PM_10_ and O_3_ in the open air. Similarly, Juneng et al. [19] analyzed PM_10_ concentrations all over Malaysia using rotated PCA. Moreover, PCA, in combination with an enrichment factor, has successfully been implemented in the assessment of the air quality of an indoor charcoal cooking restaurant; it identified the particle fraction of PM_2.5_ as a possible source of pollution [20]. Therefore, in the present study, PCA is used to identify major pollutant sources present in the mine environment.

Recently, ANN has shown great potential in the fields of engineering, industrial process control, medicines, computing, risk management, and marketing [21]. Several air quality studies [10,22] have utilized ANN to simulate PM_10_ concentrations, air quality prediction, and other environmental issues. These applications clearly indicate the high capability of ANN to accurately predict in complex environments. Cigzoglu and Kisi [23] accurately predicted air pollution in Istanbul, Turkey using feedforward backpropagation combined with a radial basis function algorithm. In regard to the mining engineering, ANN is not a new concept. An initial example for the adoption of ANN [24] in the mining industry is the real-time control of mineral processing plants. Edwards et al. [25,26] collected data from a smoke sensor installed in a mine to accurately identify the combustibles present in the mine environment. Similarly, Karacan [27,28] conducted a series of modelling, simulation, and real experiments using a neural network to accurately predict the methane gas concentration and automatically control mine ventilation. For air quality in mines, Dixon et al. [29] applied ANN to the gas monitoring data and forecasted the concentration of methane gas inside the mine environment. Similarly, Park et al. [30] simulated ANN for the prediction of the PM_10_ concentration in metropolitan subway stations in Seoul. They found the prediction accuracy of ANN to be between 60–80% relative to measured values. They also described the effect of the architecture and depth of subway stations on the ANN results. Conclusively, ANN is a valuable technique for enhancing safety in mines through its ability to predict air quality and allow the automatic control of mine ventilation. A member from the family of ANN is multi-layered perception (MLP); this has proved its ability for prediction using time series [31]. It enables easy extraction of precise information from complicated databases. MLP has shown great potential for solving complex environmental problems. For instance, Ramedani et al. [32] used relative humidity, temperature, sunshine duration, and amount of precipitation as input variables of MLP-ANN for predicting global solar radiations. Thus, it can be expected that ANN, including MLP, will be able to provide accurate prediction of air quality in the complex environment of UCMs.

## 3. Proposed System Design

### 3.1. System Architecture

**Figure 1:**
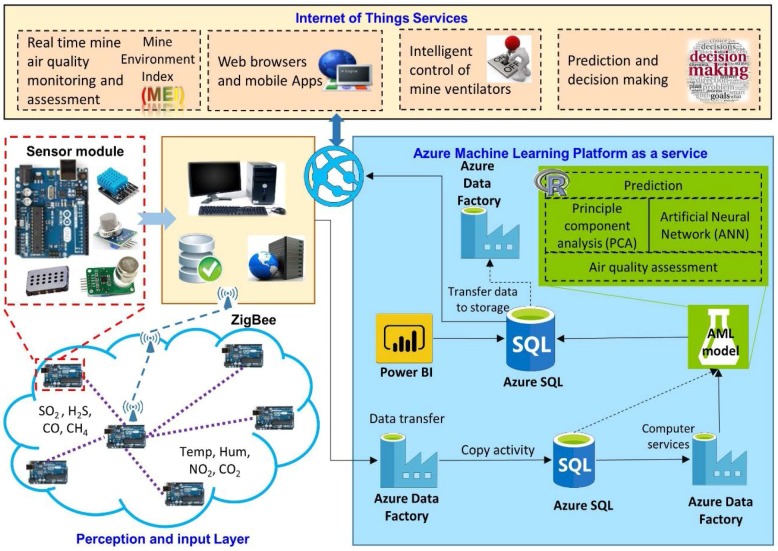
System Architecture comprising Arduino as a microcontroller for air quality monitoring in mines, based on Azure Machine Learning (AML), for air quality prediction.

The proposed system has been specifically designed for air quality monitoring and assessment in UCMs. Figure 1 shows the basic architecture of the proposed system. The main frame comprises data acquisition, data transmission, data processing for air quality assessment and prediction, and finally, services for information sharing and intelligent control of mine ventilators. In this system, sensing units are based on sensor modules attached to an Arduino UNO (ATmega1280, Atmel, San Jose, CA, USA) [33]. Two sensing units make up the sensor nodes (SNs), which capture air quality related data and transmit this data to the base station via ZigBee. The base station runs AML, which operates as a platform as a service (PaaS). The air quality model extracts pollutant types and predicts air quality depending upon the concentrations of pollutants. Thus, this system enables AML-based decision making and autonomous control. Details of each component of the proposed IoT system are described in upcoming sections.

### 3.2. System Hardware

**Sensor Node:** The basic function of a SN is to sense and measure air quality parameters inside the mine environment. Usually, SNs are comprised of sensor modules, a microcontroller, and wireless transmitters. In this study, SN is based on an Arduino UNO microcontroller. Arduino is an open-source, low-cost, and low-power controller which is run without interfacing with a computer because of its ability to load script [34]. It has 2 kB of RAM and 32 kB of program memory and operates at 5 V DC. Arduino UNO can be easily programmed into the Arduino development environment (IDE). 

**Figure 2:**
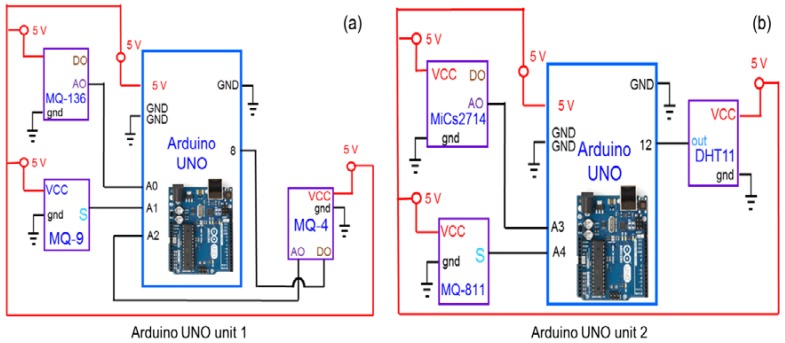
Circuit diagram of each sensor modules attached to the Arduino UNO based sensor nodes (SN) (**a**) attached MQ4, MQ136, and MQ9, (**b**) attached MiCs2714, MQ-811, and DTH11.

Selection of appropriate sensors for monitoring a mine environment is a relatively complicated issue and it demands consideration of several factors, such as measurement range, accuracy, and sensitivity. The operational temperature range of DTH11 is from −40 °C to 120 °C with ±2 °C and it has the ability to measure humidity of 20–80% with ±5% accuracy. It operates at 3.3–5 V; 2.5 mA is the maximum current usage. Usually, the temperature in a working coal mine varies between 15 °C and 45 °C, and the humidity lies within the specific range of DTH11. These potentials make DTH11 highly suitable for its use in UCMs. Common gases found in UCMs are CH_4_, CO_2_, CO, NO_2_, H_2_S, and SO_2_. This study utilizes MQ-4, MQ-9, MQ-811, MQ-136, and MiCS-2714 sensor modules to monitor the concentration of various gases. Among these, most of the sensor modules are metal oxide (tin oxide (SnO_2_)) based and respond well to volatile gas molecules; thus, they are more reliable and efficient for gas monitoring. In addition, sensor modules, either for gas monitoring or for temperature measurement, are cost-effective, low-power, stable, and are environment ineffective operational clones of the Arduino board. Generally, the gas concentration and sensor resistance have following relationship:

(1) $$R_{s}=A(c)-\alpha$$

where *R_s_* is the sensor resistance, *A* is a constant, *c* is the gas concentration and α is the slope of the *R_s_* curve. Table 1 summarizes the detection principles and specifications of each sensor module. The circuit diagram of the sensor modules attached to the Arduino UNO is shown in Figure 2. As this study has been designed to monitor eight different air quality parameters; however, the limited capacity of Arduino UNO offers a great challenge in handling large number of sensor modules. Therefore, by keeping in mind the limited capacity of the Arduino UNO and to make the proposed network economical, each SN was divided into two units. Each unit was mounted and programmed with XBee shield and a specific number of sensor modules.

**Figure 3:**
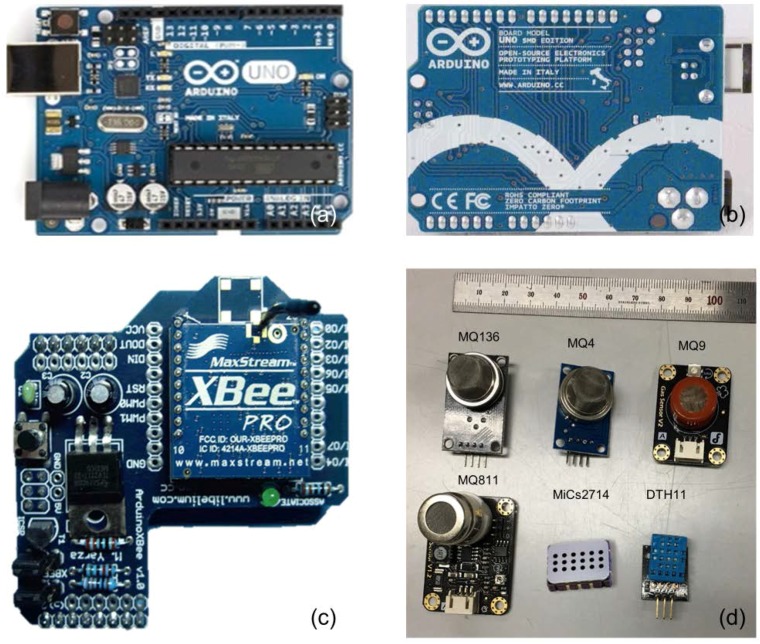
(**a**) Front pictorial aspect of Arduino UNO, (**b**) back-side pictorial aspect of Arduino UNO, (**c**) 2.4 GHz Zigbee shield for Arduino, and (**d**) visual aspect of MQ136, MQ4, MQ9, MQ811, MiCs2714, and DHT11.

**Communication Protocol:** The ZigBee wireless communication protocol has been previously shown to have high transmissivity, stability, ultra-low power, and high communication in underground mines [35]. Therefore, this study used the ZigBee 802.15.4 protocol with a bandwidth of 2.4 GHz. The XBee module (24.38 mm × 27.61 mm) was connected in series with Arduino UNO. This module has a nominal range of 30 m in open air. The ZigBee data throughput was 250 kbps, and it had a receiver sensitivity of −92 dBm, with an error drop-down transmission of ±25. Generally, longer and narrower dimensions of mine tunnels impose difficulties for WSN topology. This study uses cluster topology, in which each cluster is comprised of number of SNs and a cluster head. Figure 3 shows the visual aspects of Arduino Uno, Xbee module, and sensor modules.

**Base Station:** The base station functions as a data logger; it collects the raw data, arranges it and pushes the arranged data to AML. In this case, the base station is a PC server (Intel^®^ Xeon E5420 2.5 GHz with 8 Gb RAM) with Windows 7 (Microsoft, Redmond, WA, USA) as the operating system.

### 3.3. Azure Machine Learning for Cloud Computing

**Figure 4:**
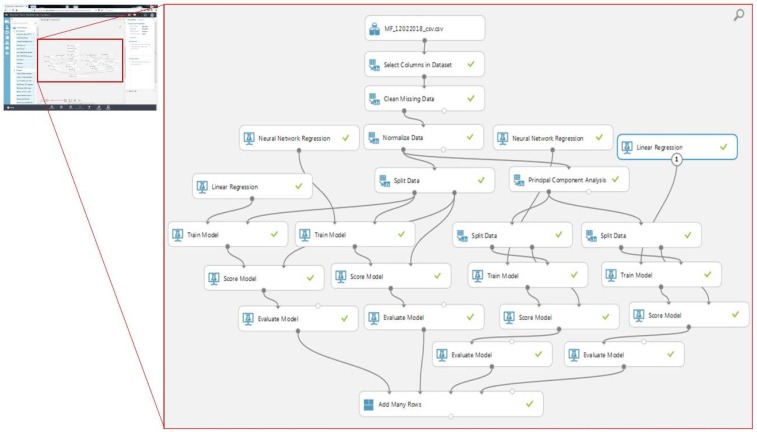
Prepared artificial neural network (ANN) model in Azure Machine Learning (AML) Studio.

Cloud services in combination with machine learning (ML) are constantly helping organizations to grow their businesses by providing massive storage and data processing ability to allow the discovery of useful patterns and trends. With the enormous number of growing datasets, the traditional libraries for the ML are becoming insufficient. Therefore, the present study utilized AML for the development, training, and testing of the mine air quality ANN model. AML is the R programming, based on an open, drag-and-drop and collaborative platform, mainly used by IT professionals, developers, and the public. It is a comprehensive set of cloud computing services equipped with learning modules, data pre-processing, statistical tools, a SQL server, and an API to launch the model in the application [36]. The main objective of adopting this ontology is to provide a simple method for data storage, computation, streaming, and sharing services for diversified IoT applications. The screenshot of the prepared model is shown in Figure 4.

## 4. Methods and Models

### 4.1. Mine Environment Index (MEI)

Various indices, such as the air quality index (AQI) [37], air pollution index (API) [38], and indoor air quality index (IAIQ) [39] have been introduced as key tools for easy and quick assessment of air quality in various environments and to predict pollutant concentrations. Among these indices, AQI, introduced by United Stated Environmental Protection Agency (US-EPA), is the most widely adopted index for the representation of open air environments. The constitutive components of this index are CO, SO_2_, PM_10_, O_3_, and NO_2_, which are commonly present in open air. As people spend 90% of their time in indoor environments [40], therefore, some researchers have also introduced indoor air quality indices. Compared to open air and indoor ambience, the environment in underground coal mines is relatively harsh, confined, and toxic because of the presence of gases such as CO, CO_2_, CH_4_, SO_2_, NO_2_, and H_2_S, which are emitted from coal beds during excavation. Thus, the indices defined for open or indoor air quality are insufficient to fully represent underground mine air quality. There should be an index available that gives a true representation of the mine environment and can readily assesses mine air quality.

However, despite extensive research on ventilation systems for underground mines, the mining industry and underground structures still lack such an index for the true representation of air quality. Therefore, for quick assessment and easy interpretation of mine air quality, this study introduces the mine environment index (MEI). This index is coined from two individual indices: the mine air quality index (MAQI) and the thermal comfort index (TCI). MAQI relies on the concentration of air pollutants, while TCI is mainly concerned with comfortable working conditions, such as temperature and humidity. MAQI has been assigned a weighting of 0.7 because of its major contribution to the mine environment, and a weighting of 0.3 has been given to TCI. Thus,

(2) MEI=0.7(MAQI)+0.3(TCI)

MAQI has been defined in a similar manner as to AQI, but it has different variables. Its representative equation is the same as that of AQI for open-air and is given as

(3) $${\mathrm{MAQI}}_{p}=\frac{\left({\mathrm{MAQI}}_{\mathrm{Hi}}-{\mathrm{MAQI}}_{\mathrm{Lo}}\right)}{\left({\mathrm{BP}}_{\mathrm{Hi}}-{\mathrm{BP}}_{\mathrm{Lo}}\right)}\times (C_{p}-{\mathrm{BP}}_{\mathrm{Lo}})+I_{\mathrm{Lo}}$$

where, MAQI_P_ is the index value for pollutant p, C_P_ is the input concentration of a given pollutant p, BP_Hi_ is the higher breakpoint that is ≥C_P_, BP_Lo_ is the lower breakpoint that is ≤C_P_, MAQI_Hi_ is the index breakpoint value corresponding to BP_Hi_, and MAQI_Lo_ is the index breakpoint value corresponding to BP_Lo_. The MAQI values have been categorized into five status categories: very good, good, moderate, poor, and very poor. The limiting values for each category are summarized in Table 2.

Regarding its application in mines, the TCI is the thermal comfort level of miners, and it is highly dependent on the ambient temperature and humidity. It can be given as

(4) $$\begin{array}{l} \mathrm{TCI}=-42.379+(2.0491523\times T)+(10.14333127\times rh)-(0.22475541\times T\times rh)-\\ (6.83783\times 10^{-3}\times T^{2})-(5.481717\times 10^{-2}\times rh^{2})+(1.22874\times 10^{-3}\times T^{2}\times rh)+\\ \left(8.5282\times 10^{-4}\times T\times rh^{2})-(1.99\times 10^{-6}\times T^{2}\times rh^{2}\right) \end{array}$$

where, *T* is the temperature measured in °F and rh is the relative humidity. The breakpoints for MEI are summarized in Table 3.

### 4.2. Data Pre-Processing

Prior to implementing any statistical approach, it is necessary to pre-process the collected data so that substantial characteristics of the sensors’ responses can be extracted to produce features for further processing. In the present study, transformation, as an initial step of pre-processing, was carried out using *z*-scale transformation with a mean of 0 and a standard deviation of 1, given as

(5) $$z_{ij}=(x_{ij}-\mu )/\sigma$$

*z_ij_* is the *j*th value of variable *i*, *x_ij_* is the *j*th observation of variable *i*, *μ* is the mean and *σ* is the standard deviation. *z*-scale transformation ensures the equal weight of variables for any statistical process. This transformation also homogenizes the distribution variance and reduces the probability of any error arising because of the different sizes of data sets [43]. Finally, the sphericity and correlations between various air pollutants were determined using Bartlett’s test with a high significance level of (*p* < 0.0001) and a threshold limit of 0.5.

### 4.3. PCA Modelling

One of the most prevailing and valuable statistical approaches is PCA, which compresses and transforms m-dimension data into a new dataset of n-dimensions, where *n* < *m*. It uncovers the potential structure of the set of variables without losing important information. PCA transforms a large data set of interrelated variables into new and uncorrelated variables, known as principal components (PCs). The PCs are orthogonal, uncorrelated and have a linear relationship with variables of the original dataset, given as

(6) $$PC_{p}=w_{1p}x_{1}+w_{2p}x_{2}+...+w_{np}x_{n}$$

where the notation for the *p*th PC for the overall n number of data is *PC_p_*, *W_np_* is the regression coefficient weight determined by PCA, and *x_n_* is the adjusted matrix. The extraction of PCs usually occurs in the gradual decreasing order of their variance. In order to determine the optimal number of PCs, several approaches exist: Broken Stick rule, Velicer’s partial correlation procedure, Cattell’s scree test, and cumulative percentage of variance. More specific to the air quality problem, Kaiser’s criteria [44] of eigenvalue-one is the most commonly used method for the selection of optimal numbers of PCs. PCA has shown a high capability for identifying the most significant air pollutant. Therefore, this study used PCA along with Kaiser’s approach to determine the most significant pollutant present in the mine environment.

### 4.4. MLP-ANN Modelling

ANN is the most widely accepted information processing system in artificial intelligence, intended as a generalized mathematical model of the brain system [45]. ANNs are made up of several interconnected neurons and have the capability to change their structure based on internal or external data. ANN can be trained for non-linear and complex data, which makes it highly suitable for the faster interpolation of predictions, clustering, and classification, as compared to digital computer systems. The prediction results in ANN are highly dependent on the number of input variables and assigned weights of each neuron [46]. A few studies [47] have reported the use of PCA outputs as input variables for MLP-ANN and have proved the validity of this approach in decision making. Similarly, in this study, the outputs of PCA were used as inputs for the MLP-ANN model to predict the air quality accurately. A MLP-ANN network was fed with PCA results for the identification of pollutant sources that significantly affect the MEI.

**Figure 5:**
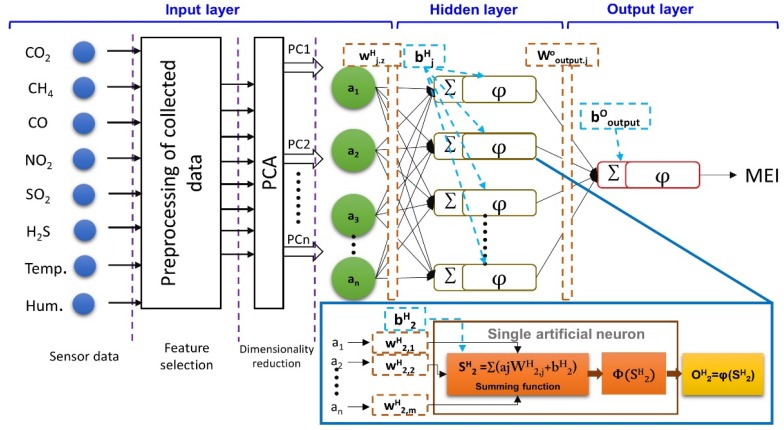
Network architecture of the multilayer perception-artificial neural network (MLP-ANN) for the mine environment index (MEI).

In the network of MLP-ANN, the first layer is the input layer, responsible for the information collection, error removal, and transmission of data to the ANN structure. The second layer is the hidden layer, with arbitrary numbers of neurons and several layers. The number of hidden layers in the present study was set to two. In this network, the neurons activate during the functions of feedforward and backward propagation providing the connections between the layers. Each neuron interacts with other neurons depending upon its ability to make connections. Based on the interaction of neutrons, each neuron is assigned a weight. The most common interactive function is the sigmoid function for the transmission of information between layers. Hidden layers receive and transmit the data between input and output layers. The hidden layer hands over the data to the next layer until the output layer is reached, to provide the output. Finally, the process outputs, after processing the collected data, are provided to the output layer through nodes in each layer connected to each other. The used MLP-ANN architecture with inputs from PCA to determine the MEI is shown in Figure 5.

The accuracy and training capacities of the MLP-ANN model are highly dependent on the selection of the optimum number of neurons. If more neurons are present, the model will be over fitted and converge quickly; if too few neurons are present, the model will be less accurate and not trained properly [48]. In the present case, the MLP-ANN model was designed in AML with weights determined by the randomization function. The model was trained using 70% of the entire data set using the Levenberg-Marquardt (LM) training algorithm [49], and the remaining 30% of the data set was used for testing. This training algorithm was adopted because of its high speed, high efficiency, and because it precisely trains the network with a standardized range: 0–1. For model learning, a widely accepted approach for environmental studies, gradient descent with momentum back propagation (0.5), was adopted. A goal of 0.001 for the mean absolute error (MAE) or 1000 epoch was set; training was continued until the model fulfilled either of these conditions. Thus, MAE and epoch numbers were the stopping criteria for training. The same process was repeated by varying the number of neurons to determine the optimal number of neurons in the hidden layer. The characteristics of MLP-ANN training are summarized in Table 4.

The optimum number of hidden neurons were determined with a hit and trail approach. The output values determined in the forward phase were transmitted back from the hidden layer, and the weights of each node adjusted themselves accordingly to minimize error, relative to original values. The performance of MLP-ANN was monitored using MAE, root mean square error (RMSE), relative absolute error (RAE), relative square error (RSE), and coefficient of determination (*R*^2^).

(7) $$\mathrm{MAE}=\frac{\sum_{i=1}^{N}\left|y_{\exp}-y_{cal}\right|}{n}$$

(8) $$\mathrm{RMSE}={{\left(\frac{1}{N}\sum_{i=1}^{N}(y_{\exp}-y_{cal}\right)}^{2})}^{0.5}$$

(9) $$\mathrm{RAE}=\frac{\sum_{i=1}^{n}\left|y_{\exp}-y_{cal}\right|}{\sum_{i=1}^{n}\left|\bar{y}-y_{cal}\right|}$$

(10) $$\mathrm{RSE}=\frac{\sum_{i=1}^{n}{\left(y_{\exp}-y_{cal}\right)}^{2}}{\sum_{i=1}^{n}{\left(\bar{y}-y_{cal}\right)}^{2}}$$

(11) $$R^{2}=\frac{\sum_{i=1}^{n}{\left(y_{pi}-y_{om}\right)}^{2}}{\sum_{i=1}^{n}{\left(y_{pi}-y_{om}\right)}^{2}+\sum_{i=1}^{n}{\left(y_{oi}-y_{pi}\right)}^{2}}$$

where *y_oi_* is the observed value, *y_pi_* is the estimated value, and *y_om_* is the average of observed values.

## 5. Calibration of Sensors and Sensor Nodes

In this study, all the sensors were calibrated in ambient air conditions, but our discussion is limited to the CO_2_ sensor. Calibration of each gas sensor was carried out on a laboratory scale under normal environmental conditions (25 °C) with the help of a sealed gas test box, SR3 (235 mm × 180 mm × 210 mm). This box can be air tightened, and it has been specially designed for the calibration of gas sensors. Before calibration, the box was opened in normal air conditions and an attached mixing fan was turned on for 4 min. Afterwards, the box lid was tightened, and CO_2_ was introduced into the box with the help of a syringe. The syringe was filled with 99.95% pure CO_2_, extracted from the gas cylinder using a syringe adaptor. After injecting CO_2_, the mixing fan was turned on for 1 min. Before recording the readings from the sensor, a time lapse of 45 s was given, as the response time of the CO_2_ sensor is 30 s. The sensor readings were recorded for 2 min, and the lid was opened in conjunction with turning on the mixing fan for 3 min.

**Figure 6:**
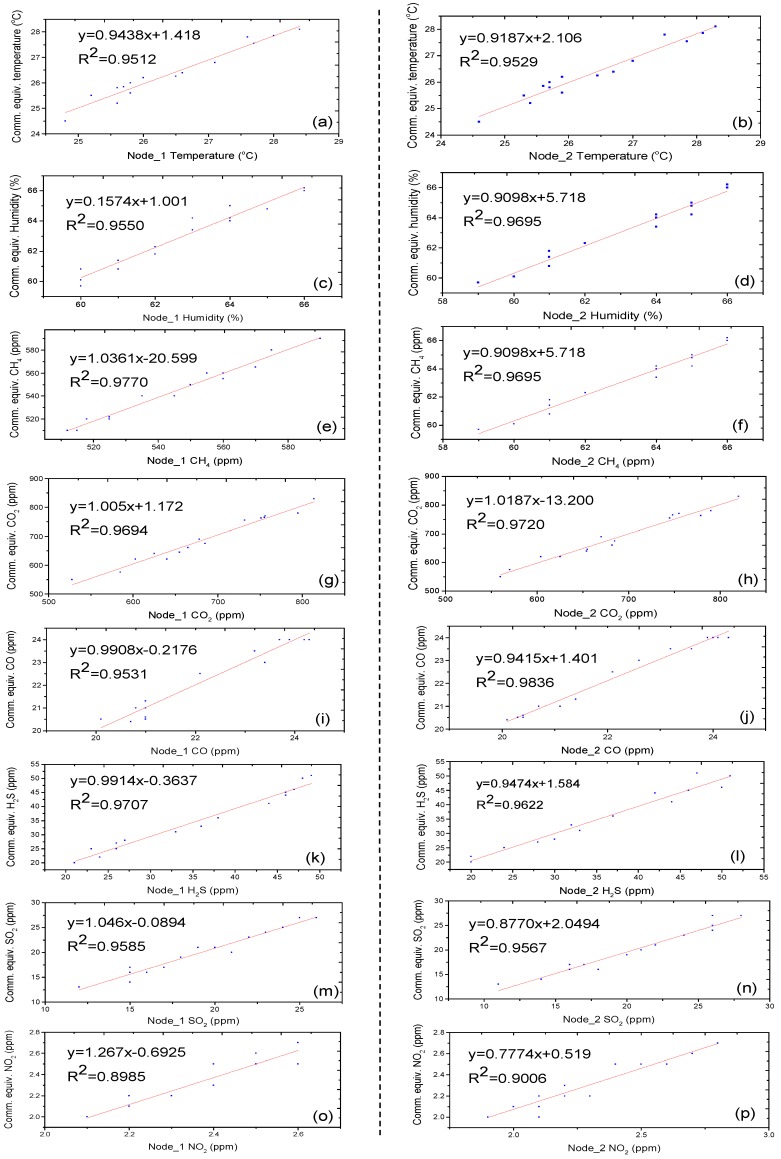
Correlation and regression plots of sensor nodes 1 and 2 for the attached sensor modules, (**a**,**b**) temperature, (**c**,**d**) humidity, (**e**,**f**) CH_4_, (**g**,**h**) CO_2_, (**i**,**j**) CO, (**k**,**l**) H_2_S, (**m**,**n**) SO_2_, and (**o**,**p**) NO_2_, in comparison to their commercial equivalents.

The Arduino-based developed SNs were validated using a commercial instrument Aeroqual-900 (Aeroqual, Avondale, Auckland, New Zealand) with additional sensors of temperature and humidity. For this purpose, the prepared SNs and commercial equivalents were placed in a glass sealed container of 170 cm× 90 cm× 50 cm at normal room temperature (25 °C). The readings were recorded for more than three hours and regression plots of sensed data in comparison with commercial equivalents are shown in Figure 6. Regression plots of all gases showed a linear relationship between the injected concentration of gases and the readings shown by the sensors. In most of the cases, the coefficient of determination was found to be greater than 0.95, except in the case of the MiCS-2714 sensor (0.90), which was still in the acceptable range. The means and standard deviations of the SNs along with their commercial equivalents are summarized in Table 5. The table demonstrates that the means and standard deviations of almost all of the parameters are comparable. These values of means and standard deviations indicate that the developed nodes have same responses as those of commercial equivalents; therefore, the readings from prepared nodes are reliable for use in UCMs.

## 6. Test Bed

**Figure 7:**
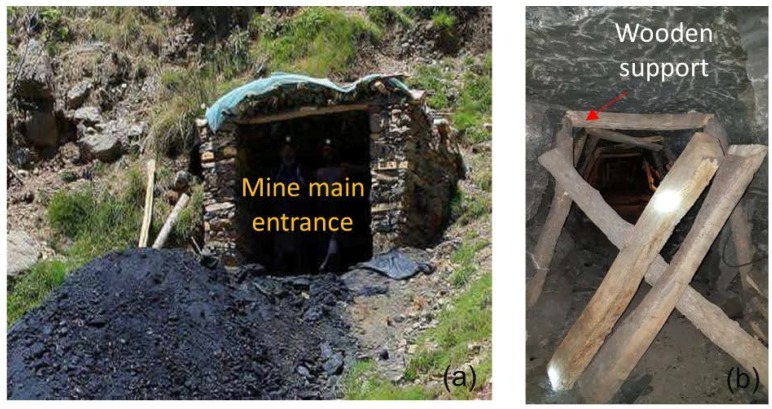
(**a**) Mine portal opening, (**b**) a closed entry of the mine with installed wooden support.

The mine tunnel used for implementation of the current system is the main roadway of an operating UCM. The dimensions of these mine tunnels were 1.8 m × 2.12 m, and almost all tunnels were supported with wooden planks at spacings of 1 m (Figure 7b). The units of SN programmed for XBee shield and sensor modules were attached to the rooftop center of the mine tunnel opening. Data transmission from both units was synchronized, based on the consensus synchronization algorithm. All of the SNs were attached to cluster heads via the ZigBee protocol. Finally, the sensed data were collected at the base station installed at the mine office. This base station arranges all the data into the specific format required by the AML. The AML model pre-processes the data to determine any missing values, trains itself by splitting the data, and then predicts the air quality.

The system’s installation was completed on 26 July 2016. Its first time start up took 90 s, and 1.5 kHz was the refresh rate. For the initial two days, the data collection rate was set at 15 min. Thus, after two days, there were 3104 samples collected, with 97 readings for each sensor per day. In the entire dataset, there were only 15 missing values, indicating the reliability of the data collection method. Among this data set, 2484 readings were used to train the model, and testing was carried out on 605 samples. At the completion of training and sensor evaluation, an extensive monitoring program was started on 29 July 2016 at midnight. Currently, this system predicts MEI recalling datasets from longer than a month.

## 7. Results and Discussion

### 7.1. Air Pollution Source Identification

**Figure 8:**
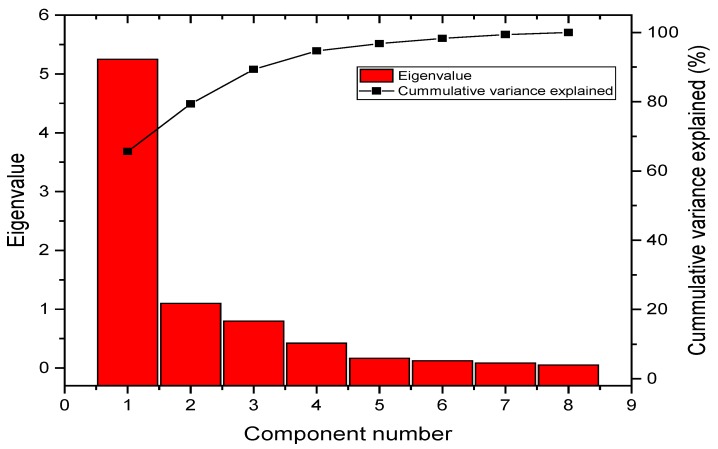
Scree plot for PCA with normalized eigenvalues.

The observed value of chi-square, 7.9 × 10^2^ (*p* < 0.05, *df* = 28), obtained from Bartlett’s test showed that the air quality test meets the sphericity assumption. This test also indicated that the variables were correlated and not orthogonal; thus, PCA can allow the interpretation of the involved parameters with a much lower number of components, compared to the original number of variables. The input to PCA was sensor readings of eight different variables. PCA extraction gave principle components (PCs). The selection of the most significant components from these eight PCs was carried out on the basis of having an eigenvalue greater than 1. At this stage, the components having eigenvalues less than 1 were neglected because of being redundant to more important factors. The eigenvalues and percentages of variance of each extracted component are summarized by Table 6. This table and scree plot (Figure 8) clearly indicates that the first two components have eigenvalues greater than 1 and their cumulative variance is 79.370%, whereby, 65.61% was explained by the first component only, and 13.76% was explained by the second component. Therefore, it is easy to say that the most effective component is the first PC followed by the second based on their eigenvalues.

In order to completely understand and to accurately interpret the data, PCs were rotated using varimax rotation. Table 7 shows the eigenvalues of PCs after rotation. The relative significance and components’ structures have been optimized with the adoption of rotation. After rotation, the variance percentages of the first PC reduced by more than 3%, from 65.610% to 62.894%. On the contrary, the variance percentages of the second PC increased, indicating the major contribution of the first PC; this shows that the PC1 is more correlated with the data relative to PC2.

In this study, the vari-factors with absolute values greater than 0.85, indicating relatively strong loadings, were set as threshold limiting values. Table 7 indicates five out of eight components which satisfy the condition of the 0.85 threshold limit value. These components are the major pollution factors in the air of this mine. These pollutants are CH_4_, CO_2_, SO_2_ and CO along with temperature. In these pollutants, PC1 contributes 65.16% to the loading factors of temperature, CH_4_, CO_2_, CO, and SO_2_. These influencing factors are mostly related to the common gases present in mine environment of UCMs. Among these gases, CH_4_ and SO_2_ are generally confined by the coal bed in the form of gas pockets. During the excavation of coal, these pockets burst out and release gases into mine air. The major cause of CO_2_ in the mine environment may be because of the breathing activity of workers as well as the exhaust from any diesel operated machinery. On the other hand, CO is only the loading factor with a contributing factor from PC2. Table 8 shows the correlations of both selected PCs.

### 7.2. Prediction Results

In order to develop the MLP-ANN model, network structures with various numbers of neurons were tested, using both original and PCA extracted components as inputs. In these MLP-ANN structures, the optimum number of neurons were determined based on MAE, RMSE, RAE, RSE, and *R*^2^. The number of neurons was gradually increased, one-by-one, and errors were determined against each number of neurons. In the case of the original data sets, trials were initially started with eight neurons and this number was increased until the minimum number of errors was indicated. In the case of PCs, the trials were initially started with three neurons which were gradually increased to determine the optimum number of neurons. In the MLP-ANN process, the sigmoid transfer function provided the optimal activation function. A validation model with different numbers of neurons for both original and PCA extracted components as inputs is summarized in Table 9. For the original data set, the optimum number of neurons to give the minimum error was 18, and for the PCA extracted data sets, the optimum number of neurons was 6.

The performance of the proposed model was compared with models defined by multi-linear regression (MLR), PCA-MLR, and ANN. For all of these models, the MAE, RMSE, RAE, and RSE were compared, as the error values close to zero indicate a better model. On the other hand, the accuracy of the model was checked by calculating the coefficient of determination (*R*^2^). In these tests, the value of the model with high accuracy was close to one. Hence, the accuracy of model can vary depending upon the time interval required for prediction. In the case of underground mines, there are several limitations; therefore, it is necessary to predict gas concentration. The best prediction model was PCA-ANN, with MAE, RMSE, RAE, and RSE having values of 0.1519, 0.2104, 0.7619 and 0.6818 respectively. Moreover, the coefficient of determination, *R*^2^, was 0.6654; this is illustrated in Table 10.

PCA improved the accuracy of the linear regression model approximately by 2.1%, and 16.9% in the case of ANN. The results show that the PCA in combination with MLP-ANN improves the prediction accuracy of mine air pollutants. Moreover, the PCA extracts important information about the major pollutants present in the mine environment. Thus, the application of PCA is helpful for prediction studies specifically related to air quality.

## 8. Conclusions

In recent years, the computing power of cloud services has revolutionized the solution of complex and nonlinear data problems. Air pollutants present in UCMs have always showed non-linearity, ultimately causing uncertain predictions and lower reliability for early warnings. Therefore, this paper introduced an IoT-based mine air quality monitoring, assessment, and forecasting system that utilizes AML cloud computing to predict air quality and has the potential to widely enhance underground mine safety through early warnings. The system was installed in an operating underground coal mine, and it advocated an air quality assessment model to determine MEI.

The following are the conclusions:

(i) In this system, IoT-sensors were used to monitor mine environment related parameters, and the limiting values of each parameter were defined separately to determine the mine’s air quality in terms of MEI. The calibration of the prepared SNs with regression constants was always greater than 95% for almost every parameter, which confirmed the reliability of system.
(ii) Two different MLP-ANN models were designed in AML studio: one for PCA applied outputs to the dataset and other one was for original dataset.
(iii) This system effectively alleviated the non-linear behavior of mine environment variables, such as temperature, humidity, CO_2_, CH_4_, CO, SO_2_, and H_2_S by pre-processing the sensors’ data. The parameters’ data was uploaded into AML studio as input data sets to the MLP-ANN model. Bartlett’s test confirmed the co-relations and non-orthogonality of the monitored variables. The PCA results indicated four mine gases (CH_4_, SO_2_, CO, and H_2_S) as having the most significant influences on mine air quality. Multi-layer perceptron from the family of ANN accurately predicted the MEI. As the accuracy and efficiency of the MLP-ANN model is highly dependent on the input parameters and hidden layer neurons numbers, the optimum number of hidden layer neurons was determined by observing minimum error.
(iv) The proposed ANN-PCA model is 14.8%, and approximately 3%, more accurate, compared to linear regression and ANN models, respectively. This study suggests that an appropriately trained MLP-ANN model can effectively forecast MEI. Moreover, Azure Machine Learning enabled quick data processing with easy web service, based on an easy graphical user interface.

Despite the test results indicating accurate forecasting, some limitations in this study still exist. These limitations are the harsh environment of underground mines, data privacy, and the integration of multi-sensors’ outputs. Moreover, the present study only considered eight air quality parameters and ignored parameters which may affect the mine environment more severely. This study relied on the concentrations of air pollutants over time, while the other factors related to the forecasting effectiveness were ignored. We propose the following future directions: Firstly, determination of the complex non-linear behavior of pollutants’ concentrations demands a more precise hybrid model which would enhance early-warnings. Secondly, high pollutant concentrations are the major contributing factors to air quality; therefore, development of a model for forecasting peak air pollutant concentrations is required.

## Figures and Tables

**Table 1:** Sensors used for experimentation and their characteristics.

Characteristics	Sensor Module					
	MQ9	MQ4	MQ811	DTH11	MQ136	MiCs2714
Sensor type	MOS	MOS	MOS	Negative Temperature coefficient (NTC) sensor	Metal Oxide Semiconductor (MOS)	MOS and Microelectro-mechanical systems (MEMS)
Target gas	CO	CH_4_	CO_2_	Temperature and humidity	SO_2_ and H_2_S	NO_2_
Typical detection range	10–1000 ppm	200–1000 ppm	350–10,000 ppm	20–90% RH *, 0–50 °C	0–100 ppm (SO_2_ and H_2_S)	0.05–5 ppm
Accuracy	±5%	±5%	±5%	±5% RH, ±2 °C temperature	±5%	±5%
Sensitive material	SnO_2_	SnO_2_	SnO_2_	--	SnO_2_	--
Sensitivity	High	High	High	±0.7 °C	3 ppm	0.25 ppm
Respond time	20 s	20 s	20 s	6 s (hum) and 10 s (temp)	30 s	30 s
Manufacturer	Hanwei electronic Co.	Hanwei electronics Co.	Hanwei electronics Co./Sandbox electronics	Aosong electronics Co.	Microelectr-onics Ltd.	SGX sensor Tech.

* Relative humidity.

**Table 2:** Breakpoints of various gases for mine air quality index (MAQI) and its categories [41,42].

Condition	Gases (ppm)					
	NO_2_	CO	SO_2_	H_2_S	CH_4_	CO_2_
Very good	0–1	0–12	0–2.5	0–3	0–1000	0–2000
Good	1.1–2.0	13–22	2.6–4.0	3.1–5	1001–2000	2001–3000
Moderate	2.1–3.0	23–30	4.1–6.0	5.1–12.9	2001–4000	3001–4000
Poor	3.1–4	31–49	6.1–8.0	13–20	4001–5000	4001–5000
Very poor	>4	>50	>8	>20	>5000	>5000

**Table 3:** Break points for the mine environmental index (MEI).

MEI	MAQI	TCI
0–50	Very good	Mostly comfortable
51–100	Good	Comfortable
101–200	Moderate	Neutral
201–300	Poor	Discomfort
301–500	Very poor	Least comfort

**Table 4:** Training parameters for the multilayer perceptron (MLP) model.

Training Parameters	Value
Sample: Training samples: 2484 Testing samples: 605 Missing values: 15	3104
Input parameters	8
Hidden neurons	Flexible
Output neurons	1
Performance	MAE, RMSE, RAE, RSE, R^2^
Goal	0.001
Learning rate	0.01
Momentum constant	0.5

**Table 5:** Mean and standard deviations of nodes for gas module calibration.

Parameters	Node No. 1		Node No. 2		Commercial Equivalent	
	Mean	STDEV	Mean	STDEV	Mean	STDEV
Temperature (°C)	26.42	1.09	26.4	1.12	26.36	1.06
Humidity (%)	62.7	2.08	62.93	2.31	62.98	2.13
CH_4_ (ppm)	545.66	23.54	543	25.99	544.8	24.68
CO_2_ (ppm)	684.46	81.76	689.46	80.78	689.2	83.47
CO (ppm)	22.24	1.49	22.14	1.59	22.25	1.51
H_2_S (ppm)	35.6	10.44	35.2	10.88	34.93	10.51
SO_2_ (ppm)	19.2	4.22	20.46	5.04	20	4.52
NO_2_ (ppm)	2.33	0.16	2.27	0.26	2.28	0.219

**Table 6:** Initial eigenvalues and total variance explained.

Component	Initial Eigenvalues			Extraction Sums of Squared Loadings			Rotation Sums of Squared Loadings		
	Total	Var (%)	Cum (%)	Total	Var (%)	Cum (%)	Total	Var (%)	Cum (%)
1	5.249	65.610	65.610	5.249	65.610	65.610	5.031	62.894	62.894
2	1.101	13.760	79.370	1.101	13.760	79.370	1.318	16.477	79.370
3	0.798	9.977	89.348						
4	0.424	5.305	94.653						
5	0.168	2.102	96.755						
6	0.123	1.542	98.297						
7	0.086	1.072	99.369						
8	0.051	0.631	100.000						

Var = variance, Cum = cumulative.

**Table 7:** Rotated component matrix and possible source identification.

	Component	
	1	2
Temperature	0.873	0.171
Humidity	−0.739	0.162
CH_4_	0.908	0.203
CO_2_	0.824	−0.084
CO	0.066	0.960
NO_2_	0.772	0.129
SO_2_	0.868	0.414
H_2_S	0.930	0.324

**Table 8:** Component transformation matrix for selected PCs.

Component	1	2
1	0.973	0.229
2	−0.229	0.973

**Table 9:** Model validation with various neurons based on original dataset as input and PCA extracted components as input.

Model Characteristics	No. of Hidden Nodes	MAE	RMSE	RAE	RSE	*R*^2^
Original parameters	8	0.2060	0.2876	0.6790	0.5828	0.4371
	9	0.1904	0.2776	0.6649	0.5877	0.4372
	10	0.1885	0.2727	0.6601	0.5936	0.4463
	11	0.1897	0.2688	0.6579	0.6112	0.4737
	12	0.1769	0.2633	0.6586	0.6149	0.4850
	13	0.1734	0.2628	0.6444	0.6184	0.5165
	14	0.1725	0.2576	0.6425	0.6228	0.5371
	15	0.1704	0.2541	0.6396	0.6273	0.5526
	16	0.1692	0.2506	0.6393	0.6365	0.5584
	17	0.1691	0.2448	0.6340	0.6339	0.5806
	18	0.1647	0.2430	0.6317	0.624	0.5862
	19	0.1768	0.2568	0.6457	0.6250	0.5749
	20	0.1859	0.2618	0.6333	0.6300	0.5849
PCA extracted components	3	0.182423	0.250838	0.78808	0.701039	0.608961
	4	0.169745	0.233575	0.775804	0.696784	0.633216
	5	0.161998	0.223779	0.763809	0.687294	0.652706
	6	0.1519	0.2104	0.7619	0.6818	0.6654
	7	0.157451	0.228864	0.762694	0.695096	0.624904
	8	0.16392	0.243648	0.786798	0.702337	0.597663

**Table 10:** Performance indicators for MEI prediction models.

Model	MAE	RMSE	RAE	RSE	*R*^2^
LR *	0.1829	0.2653	0.6163	0.4547	0.5252
PCA-LR	0.179	0.240	0.6995	0.4937	0.6063
ANN	0.1647	0.2430	0.6317	0.642	0.5862
Proposed model	0.1519	0.2104	0.7619	0.6818	0.6654

* linear regression.
